# Cardiac arrest survivors – Psychiatric comorbidity and cognitive impairment

**DOI:** 10.1192/j.eurpsy.2022.1595

**Published:** 2022-09-01

**Authors:** J.M. Borg, E. Stenager, Y. Wang, L. Svendstrup Christensen, R. Goos, F. Lund Henriksen

**Affiliations:** 1 The Region of Southern Denmark and The Institute for Regional Healthresearch (IRS); Southern Danish University (SDU), The Department Of Psychiatry, Aabenraa, Denmark; 2 The Region of Southern Denmark, The Department Of Psychiatry, Aabenraa, Denmark; 3 South Jutland Hospital, The Department Of Cardiology, Aabenraa, Denmark; 4 Odense University Hospital, The Department Of Cardiology B, Odense, Denmark

**Keywords:** Cardiac arrest survivors, cognitive impairment, Psychiatric comorbidity

## Abstract

**Introduction:**

In 2019 there were 1,760 patients in Denmark’s hospitals who experienced cardiac arrest (IHCA patients = In Hospital Cardiac Arrest patients). Of these patients about 70% survived. There is only limited knowledge about the mental and cognitive state of cardiac arrest survivors. However, it seems, that cardiac arrest survivors, perform mentally and cognitively worse compared to the background population. The mental and cognitive difficulties can lead to reduced quality of life for both those affected and their relatives.

**Objectives:**

Because the above-mentioned area has limited knowledge, further studies are needed to shed more light into the problem.

**Methods:**

To find out if the patients can be included in the study, the patient journals will be studied. After that there will be performed an interview-survey-based study, in which IHCA patients’ possible symptoms of depression, anxiety, PTSD and suicide risk, the patients’ quality of life and any cognitive disorder, shortly after and three months after cardiac arrest, will be examined. The study will also, if possible, focus on the patients’ relatives and on the eventual difficulties they may experience in the aftermath of a relative surviving a cardiac arrest. The above-mentioned will be done using already existing relevant psychiatric and neuropsychological examination tools. In relation to the patients’ relatives, however, a separate survey tool, that has been developed, will be used.

**Results:**

It is an ongoing study. Results are expected in 2023.

**Conclusions:**

In the long run the study hopefully can contribute to establishing relevant help, counseling and rehabilitation for the patients and relatives affected.

**Disclosure:**

No significant relationships.

